# Exploring Parental Views of Remote Vision Testing in Children

**DOI:** 10.22599/bioj.541

**Published:** 2026-07-06

**Authors:** Kiran Pannu-Dhillon, Ahalya Subramanian, Miriam Conway

**Affiliations:** 1 City St Georges University London, United Kingdom

**Keywords:** vision screening, vision testing, school-aged children, remote assessment

## Abstract

**Background:**

The rise of digital technologies has enabled vision testing via applications on phones, tablets, and computers, allowing assessments beyond clinical settings. While these tools empower parents to test their children’s vision at home, little is known about the attitudes of parents in the United Kingdom (UK) toward this approach. This study examines parents perceived benefits and challenges of conducting vision tests for young children at home, either independently or with the help of a qualified clinician.

**Research aim:**

This qualitative study explores parents’ perspectives on at-home remote vision testing in a paediatric population.

**Methodology:**

Data was collected via two online focus groups with parents of children aged 4–8 years old. Sessions were recorded, transcribed, and thematically coded to identify key patterns and themes.

**Results:**

Five key themes emerged: navigating trust in remote vision testing, impact of child demographics, desire for professional support, digital enablers and barriers, and appointment frequency influences desire for remote testing. Parents emphasised the need for training in remote vision testing to build confidence and skill. Potential benefits included reduced travel, time off work/school, and greater flexibility. Concerns around test accuracy, occlusion, and application use highlight the need for clear guidance and virtual supervision by professionals.

**Conclusion:**

Remote vision testing could be a reliable alternative to clinic visits, provided parents receive adequate training and support. Participants expressed concerns about the reliability of remote vision testing, particularly for younger or less cooperative children, but acknowledged benefits such as reduced travel, lower costs, and greater flexibility. While app-based testing was appealing, most preferred professional involvement, either in person or virtually, to ensure accuracy, reassurance, and follow-up support.

## Introduction

National Health Service (NHS) outpatient services are adopting innovative patient interaction and monitoring methods, driven by rising eye care demand that exceeds supply and the rapid growth of accessible digital and communication technologies (Osborne *et al*., 2023). Modernising service delivery is vital to optimise NHS resources. In the NHS, 79% of paediatric eye care appointments are related to amblyopia detection and management (Stewart *et al*., 2016). This condition affects 2–5% of children in the United Kingdom (UK) (Carlton *et al*., 2008) with an estimated treatment cost of £1365 per child (Awan *et al*., 2010). The situation is compounded by the fact that up to 50% of these children do not respond well to the gold standard occlusion therapy (Awan *et al*., 2010; Williams *et al*., 2008) prolonging treatment duration. Early therapy for amblyopia is crucial (Stewart *et al*., 2007) as age significantly influences treatment efficacy. Management for conditions such as amblyopia require frequent hospital visits for vision checks (Awan *et al*., 2010). The recurrent absences from work and school, are barriers to eye care and often result in missed appointments (Parsons, Bryce and Atherton, 2021) hampering treatment outcomes.

NHS paediatric ophthalmology services are currently under significant pressure due to workforce shortages, with nearly 60% of departments reporting vacancies and many trainees discouraged from entering the specialty because of its complexity and challenges in examining children (Clough, Magan and Jain, 2025). These staffing constraints contribute to outpatient backlogs across the UK (RCOphth, 2022), which can delay treatment and increase the risk of vision impairment in children (Stewart *et al*., 2007). Remote vision testing could help alleviate service pressures by reducing the need for frequent in-person appointments, improving overall clinic efficiency (Osborne *et al*., 2023; Painter *et al*., 2021). By increasing service capacity, it may also lessen the burden on staff and reduce waiting times, addressing both patient and healthcare system needs.

Technology could potentially be used to enable vision checks ‘at home’ enhancing monitoring and management, reducing costs, and the need for frequent travel to the hospital (Painter, Hamilton and Livingstone, 2023). Remote vision testing may support ongoing vision monitoring outside the clinic, benefiting both the hospital and the patient. Several different models exist for remote testing of vision. Parents can a use a tablet device to assess their child’s vision at home, however certain barriers may hinder the process. Parents may have concerns over their capacity to accurately assess their child’s vision remotely especially if their child is of a younger age. An alternative method of carrying out remote vision testing is, clinician-led home vision testing via video conferencing (Painter *et al*., 2021). This methodology has the potential to provide better control over the exam, such as advice on testing distance, correct occlusion of each eye and allowing the clinician to view the child’s behaviour throughout testing (Painter *et al*., 2021). Consequently, there might be less barriers to this form of remote vision testing as parents perceive it to be more accurate due to the input from trained clinicians.

Remote vision testing technologies vary in approach. Some are fully automated, while others require parental or clinician guidance to ensure correct occlusion, testing distance, and response recording. Tests may assess near or distance visual acuity, and differences in protocols mean studies are not always directly comparable (Painter *et al*., 2021; Suo et al., 2022). Recognising these variations is essential when evaluating feasibility and reliability in children.

Several different studies have found that remote vision testing by parents or orthoptists demonstrates high repeatability comparable to traditional in-clinic assessments by professionals, regardless of the child’s visual status (Allen *et al*., 2021; Bellsmith *et al*., 2022; Thirunavukarasu *et al*., 2022; Xian *et al*., 2023). The mean difference in visual acuity was ≤0.15 logMAR, matching the typical repeatability of ±0.15 logMAR (±8 letters) seen in standard clinic tests across multiple visits (Manny *et al*., 2003). These findings suggest that remote vision testing is a reliable, and convenient, alternative to in-clinic visual acuity measures (Manny *et al*., 2003). Yet, parents’ perspectives on assessing their child’s vision ‘at-home’ using innovative applications (apps) on home devices is unknown in the UK. The rapid growth of digital technologies over the past decade has brought a surge in the number of visual acuity apps for devices such as computers, iPads, and smartphones (Suo *et al*., 2022). Parents in the UK already have the option to test their children’s vision at home, but their acceptance of the technology remains uncertain.

The current study investigates parental views on the feasibility of remote vision testing in children between the ages of 4–8 years old, which refers to at home visual acuity assessment conducted using digital platforms without the need for an in person clinical visit. Parents were given the option to assess their child’s visual acuity either independently at home or with online guidance from a professional. The 4–8-year age group was selected because children in this range are at the highest risk of permanent vision impairment from amblyopia if not treated appropriately (Carlton *et al*., 2008). Additionally, research from both the UK and internationally has demonstrated that remote vision testing is feasible and effective in this age group (Allen *et al*., 2021; Bellsmith *et al*., 2022; Thirunavukarasu *et al*., 2022; Xian *et al*., 2023).

### Methodology

#### Participants

Ethical approval was obtained from City St. George’s, University of London, Optometry proportionate review committee, this study was conducted as research. Written informed consent was obtained from all participants. The research adhered to the tenants of the Declaration of Helsinki. Focus groups were chosen as the primary method of gathering data (Tong, Sainsbury and Craig, 2007) owing to their ability to thoroughly examine participants’ attitudes, beliefs, and worries (Gill *et al*., 2008; Gill, 2020; Marshall, 1996). Inclusion required guardians of children aged between 4–8 years. Exclusions included optical professionals, non-English speakers, or those without consent. Twelve participants met the criteria and were included in the study.

Participants averaged 38.75 years old (range 27–45). Most were female (67%, n = 8) and had two children (83%, n = 10). Detailed demographics including occupation are provided in Table 1. Participants were recruited via email invitations sent through local school parental networks. We acknowledge that this approach relied on voluntary participation and digital access, which may have influenced the sample. Alternative methods, such as anonymous paper surveys in clinic, were considered but were not feasible within the scope of this study.

**Table 1:** Participant demographics.


PARTICIPANT	AGE (YEARS)	GENDER	OCCUPATION	NUMBER OF CHILDREN 4–8 YEARS OLD	HIGHEST EDUCATION LEVEL ATTAINED

**Focus Group 1**					

**1**	40	Female	Teacher	2	Undergraduate

**2**	42	Male	Head teacher	2	Postgraduate

**3**	44	Female	Immigration officer	2	Undergraduate

**4**	39	Female	Collections Officer	2	Undergraduate

**5**	38	Female	Customer Operation	2	Undergraduate

**6**	39	Female	Teacher	2	Undergraduate

**Focus Group 2**					

**7**	45	Male	Healthcare	1	Postgraduate

**8**	42	Female	Business owner	1	A-levels

**9**	36	Female	Paralegal	2	Postgraduate

**10**	38	Male	Self employed	2	A-levels

**11**	35	Female	Pharmacist	2	Postgraduate

**12**	27	Female	Content Creator	1	A-levels


Data was collected through two semi-structured focus groups (Santhosh, Rojas and Lyons, 2021) remotely via Zoom. A password-protected electronic link was sent to all participants. Participants were contacted before Zoom sessions to resolve technical issues. Focus group sessions were limited to one hour as it reduces fatigue and scheduling conflicts (Gray *et al*., 2020). Remote conferencing enabled broader participation by removing travel and timing barriers (Gray *et al*., 2020). Focus groups were video, and audio recorded, to capture verbal responses and body language (Santhosh, Rojas and Lyons, 2021). The facilitator introduced the session’s goal, rules, and confidentiality. To ensure objectivity, the focus group was moderated by an orthoptist (MC) with experience in qualitative research methods. Measures to minimise bias included following a structured discussion guide, maintaining neutral language, and involving a second researcher in the thematic analysis. Consent was obtained (Kadam, 2017), non-consenting participants were allowed to exit. Participants raised virtual hands to speak; the facilitator used semi-structured and probing questions. Open-ended questions elicited deeper insights (Ahmad *et al*., 2019). Participants were not required to trial specific home vision testing applications prior to the focus groups; discussions were exploratory and based on parents’ perceptions of remote vision testing following a general explanation of the concept. Participants were addressed by name during recording for psychological safety (Davies *et al*., 2020). Names were omitted during transcription to preserve confidentiality. Participants were pseudonymised with ID numbers. Data were stored securely, recordings deleted after transcription to protect confidentiality.

Transcripts were refined and analysed to identify recurring themes and key insights (Roberts, Dowell and Nie, 2019). Data were coded and thematically analysed by KPD using an inductive approach, with transcripts coded line-by-line to identify recurring patterns; with a second researcher (Agwah Nadeem Butt) who independently reviewed the coding to ensure reliability and minimise bias (Castleberry and Nolen, 2018). Member checking verified findings with participants to ensure credibility (Lochmiller, 2021).

### Results

Analysis of the focus group transcripts identified five main themes that reflect parents’ perceptions of remote vision testing for children aged 4–8 years. These themes capture key influences on acceptability, including trust in remote testing, child-related factors, the desire for professional support, digital enablers and barriers, and the impact of appointment frequency.

Table 2 presents the five main themes and associated subthemes that emerged from the analysis of focus group transcripts. These themes reflect key factors influencing perceptions and experiences of remote vision testing in children.

**Table 2:** Five main themes and several sub-themes were identified from the transcripts of the focus group discussions.


MAIN THEMES	SUBTHEMES

**Navigating trust in remote vision testing**	Parental engagement and trustworthiness of themselves or the app Potential benefits of at-home testing

**Impact of child demographics**	Influence of child demographics on remote testing

**Desire for professional support**	Desire for professional guidance Desire for gold standard testing

**Digital enablers and barriers**	Technical barriers and need for support Equity and Access Considerations

**Appointment frequency influences desire for remote testing**	Preference for virtual health professional led at home testing


#### Theme 1 – Navigating trust in remote vision testing

Participants emphasised the importance of precise and reliable testing for children’s vision, noting concerns about external factors such as lighting and test distance affecting accuracy. They stressed the need for clear guidance and supervision to ensure parents follow instructions properly.

- Participant 5: ‘I would find it quite difficult to control all the environmental factors. At home, if you have got a set level of overhead lighting.’
- Participant 6: ‘It depends on if the parents’ is doing it properly, they might be doing it at the wrong distance then the results are not accurate.’

Trust varied among participants, influenced by their confidence in technology and self-assessment ability. Many noted that professional endorsement would increase their confidence.

- Participant 5: ‘It depends on how confident you are because some people might not be as tech savvy as others.’
- Participant 12: ‘I’d be a little bit reassured if the app was created and developed by healthcare professionals.’

Participants acknowledged benefits for example, saving time, reducing travel, and flexibility, especially for families with frequent appointments or limited access to clinics.

- Participant 3: ‘For someone who has to go into hospital for vision checks every six or eight weeks… it saves them time, in the journey and waiting in the hospital.’
- Participant 9: ‘It would be helpful for working parents and so do not have to take too much time off work. Save on cost of travelling to appointment.’

Concerns were raised about missing other eye health issues without full clinical exams and parental bias in reporting results.

- Participant 4: ‘I’d rather get everything checked. It’s like having a full MOT… you don’t know if there was an underlying issue behind there that you’re not picking up.’
- Participant 2: ‘You would completely be dependent on a parent’s honesty… some parents could fabricate… I think bias could play a part.’

#### Theme 2 – The impact of child demographics

Parents reflected that the child’s age, personality and cognitive ability could influence the acceptability and effectiveness of remote vision testing; younger children might have differences in their cooperation or attention span, that could potentially affect the validity of the test results.

The variability in children’s responses was also considered. While some children might struggle or lose interest, others could engage more readily due to the novelty.

- Participant 9: ‘Some children will respond better to an outsider than they would with their parents, every child is different.’
- Participant 5: ‘Kids really love new things, so they would see it as something new and fun to try.’

The impact of age on children’s capacity to effectively engage in remote vision tests was shown to be significant. Younger children, such as two-year-olds, may have insufficient attention span or comprehension to complete the test, whereas older children, such as four-year-olds, may find it engaging.

- Participant 5: ‘my two-year-old if the test involved letters and numbers, he’d be fidgety.’ Compared to ‘my four-year-old would find it interesting like oh this is cool, something a bit different.’
- Participant 12: ‘Using an app just to double check your child’s vision with a professional online. It is effective, depending on the child’s age and how cooperative the child is.’

Participants described the influence of children’s personalities on their willingness to engage in vision tests. Shy or easily distracted children may present challenges, while more cooperative children may find the process smoother.

- Participant 11: ‘sometimes children lie, and they are like my vision’s not great, mum. So, it’s good just to check it yourself at home.’
- Participant 7: ‘it’s the personalities and it’s also the ability of the child themselves, which are variable.’
- Participant 2: ‘I would give it a go, with a child that I know would be able to sit still and do it.’

#### Theme 3 – Desire for professional support

Despite the convenience of remote testing, participants indicated a need for support from professionals. They appreciated the knowledge and encouragement offered during in-person consultations with healthcare providers.

The value of qualified oversight was highlighted in reducing mistakes and ensuring the accuracy of the testing process when remote vision testing in children.

- Participant 12: ‘I just think if this app is going to be as effective as it should be, then it should eliminate as much error. Someone who is a professional themselves and has that qualification overlooking while using the platform.’

Participants consistently indicated a strong preference to seek advice and opinions from healthcare professionals. The desire for reassurance in the accuracy of the testing was an underlying motivation with recognition of the knowledge and experience that trained professionals provide during the assessment process.

- Participant 9: ‘I would second guess myself if I was doing a vision test on my child just because I’m not a professional.’
- Participant 6: ‘I will still be needing the validation from the healthcare professionals so that I know that what I’m doing is right for my child.’

Participants expressed a desire for follow-up consultations with professionals after completing remote vision tests, to validate test results and seek further support and guidance if needed.

- Participant 9: ‘I do like the idea if I did the test and then I was able to have a follow up with a professional to see if our scores matched.’
- Participant 6: ‘I’m going to rely on the professional opinion and if there any gaps, you know they can always refer me onwards to go to the hospital for a face-to-face appointment.’

Despite the convenience of remote testing, participants maintained a higher level of trust in traditional, in-person consultations with professionals.

- Participant 5: ‘I might be swayed a little bit because the professional is doing it online with me. I still would prefer to go to the hospital to get it checked.’
- Participant 10: ‘I would still be a nervous and anxious parent, and I would want to bring him in for a face-to-face appointment.’

#### Theme 4 – Digital enablers and barriers

Concerns were voiced about internet access, technical difficulties, and participants lack experience in this new area.

Views were expressed on new innovations and their dependability, with some participants expressing a favourable outlook on the progress of technology.

- Participant 5: ‘I’m all about innovations and technology, it has advanced so much now, it is a reliable option.’

The value of technical know-how was highlighted alongside the need for assistance in overcoming potential difficulties while utilising remote testing equipment.

- Participant 10: ‘It does depend individually, because some people are very good with technology and some people are very good at following instructions. So, in that case, a lot of people will find it quite easy to use the app if there is a video with instructions.’
- Participant 10: ‘I think if it’s a technical thing or if you’re just not confident about using the equipment, or you want to know the distance that it should be set at, it would be good to speak to someone just to reinforce everything.’

Participants commented on their concern of the potential obstacles associated with internet access and technical issues may hinder the accuracy and dependability of remote testing. Thus, face-to-face assessments were suggested since they offer higher levels of precision and reliability.

- Participant 6: ‘Interruption with our internet connection can sometimes be a hindrance so it could waste the clinician’s time. If you live in a rural location as opposed to an urban location, these are factors to consider with internet connections. It would create inequality to access depending on their location.’

Some parents indicated a greater willingness to opt for remote vision testing when it was used for general screening purposes, rather than for monitoring an existing eye condition.

- Participant 7: ‘I’d be happy to use it just to check if everything’s okay, but if my child already had an issue, I’d want a professional to keep an eye on it.’

#### Theme 5 – Appointment frequency influences desire for remote testing

Discussions focused on how often to test, weighing the advantages of remote testing for frequent check-ups against the value of in-person assessments for vision testing.

Participants voiced preference for app-based remote testing because of the convenience of regular monitoring.

- Participant 5: ‘I might not be able to get to the hospital with three monthly intervals. Using the app, I can constantly kind of check and monitor.’

Many participants opted for an online professional while utilising the application at home with their child, emphasising the benefits of both contact with healthcare professionals and remote testing at home.

- Participant 12: ‘It would be convenient to have the concept of being able to do it through an app and then having that confirmed by the healthcare professional online.’
- Participant 11: ‘If you are an anxious parent as well, if I’m worried that my child’s vision is getting worse, then I can just check myself at home and see if it’s deteriorating.’
- Participant 3: ‘If my child had to come into hospital for vision checks repeatedly every six or eight weeks. I wouldn’t personally mind doing this online with a health professional.’

## Discussion

This study is the first to explore parental views on remote vision testing for children aged 4–8 years in the UK. Parents emphasised confidence in home testing, perceived benefits, and trust in the technology. COVID-19 highlighted the need for innovation, prompting a shift to telemedical appointments for monitoring in ophthalmology clinics. During COVID-19 isolation, 1.2 million UK people used remote consultations daily, with numbers still rising (Doraiswamy *et al*., 2020). Remote vision testing is as reliable as in-clinic visual acuity tests, allowing direct comparison and tracking of results (Ritchie *et al*., 2021). Ensuring all parents feel competent in vision testing is vital. Future studies should assess training programmes to boost parental confidence and evaluate their effectiveness.

Participants noted reduced cooperation and attention in younger children, Painter *et al*., (2023) support this, noting greater variability in visual acuity due to ongoing visual development and limited cooperation in this age group. Multiple studies report a positive correlation between age and reliability in completing letter or symbol chart tests in normally functioning children (Cotter *et al*., 2015; Samanta *et al*., 2023; Qin *et al*., 2020) with several reporting that the ability to complete abstract symbol charts (Lea, HOTV, Tumbling E) in children aged 2–6 years ranges from 54% to 98.7% respectively and that the ability to complete abstract symbol charts (Lea, HOTV, Tumbling E) in children aged 2–6 years ranges from 54% to 98.7% respectively (Becker *et al*., 2002; Shallo-Hoffmann *et al*., 2004). Children under five may struggle with letter reversals, abstract symbol recognition, and orientation perception (Qin *et al*., 2020; Weitzman *et al*., 2022) skills that are required for vison assessment in commonly used charts such as Tumbling E. The accuracy of visual acuity tests in young children can be achieved by avoiding complex and time-consuming testing apps (Qin *et al*., 2020). Research suggests remote vision testing is more suitable for children over 5–6 years (Year 1), a view supported by parental feedback in this study.

Study findings suggest unstable, or absent internet access may hinder home testing, worsening the potential digital divide. The effectiveness of such technology depends on reliable connectivity and using appropriate devices. Telemedicine advances risk widening health inequalities. According to the Office for National Statistics, 96% of Great Britain households have internet access, with no significant differences in internet access, broadband speed was found between rural and urban areas (Jordan *et al*., 2004).

Participants were from moderate to high socio-economic classes, had convenient access to digital devices and did not express any concern regarding access to devices for at-home vision testing. However, access to digital devices has been highlighted by Osborne *et al*. (2023) in children from low socio-economic class or lower household incomes (Ofcom, 2020) which needs consideration when planning service provision, by policymakers (Osborne *et al*., 2023). Web browsing devices could potentially be issued or loaned as an alternative to require hospital attendance in patients who are not suitably equipped (Ritchie *et al*., 2021). Children in the lowest socioeconomic classes are 1.82 × more likely to have amblyopia (Williams *et al*., 2008). Loaning web browser devices could be a solution but would require significant investment and policy support. An example is in paediatric low vision clinics; digital devices are loaned to children after handling instruction and brief training. An alternative solution could potentially be using smartphone-based apps to improve equity of access for lower socio-economic households (Painter, Hamilton and Livingstone, 2023) as many households will have access to a smartphone but not necessarily a desktop/laptop computer or tablet which is often required to run remote vision testing apps accurately. Painter *et al*. (2023) emphasised screen rotational errors when using smartphone apps and wrong testing distances will require programs to ensure such errors are eliminated. Nonetheless, families without access to appropriate digital technology should have priority for face-to-face appointments, due to the documented link between digital exclusion and the potential risk of poor health (Allen *et al*., 2021).

Participants were more confident in apps developed or validated by healthcare professionals. Yeung *et al*. (2019) identified 42 visual acuity testing apps available but noted that only a few had undergone clinical validation. This concern is echoed by Allen *et al*. (2021), who also highlight the limited number of apps supported by robust clinical evidence. Unregulated and unvalidated vision testing apps may lead parents to use inaccurate tools not benchmarked against gold standard charts, risking incorrect vision assessments (Samanta *et al*., 2023). Paid apps can limit clinical access for some patients (Steren, Young and Chow, 2021).

Amblyopia requires visual acuity checks in a hospital approximately every six weeks, often over the course of a few years (West and Williams, 2016). Participants mentioned the many advantages of remote testing including reducing travel costs, travel time, time away from school/work, flexibility for parents, and keeping children in a familiar setting. These advantages have led to an increase in the use of telemedicine consultations in healthcare to triage and manage patients (Gerbutavicius *et al*., 2020). Conversely, a few participants noted that parents might become fixated on testing their children’s vision repetitively or concerns other pathology could be missed if relying on vision testing alone. This highlights the significance of parental training initiatives, to provide reassurance and clear instructions to parents. Some participants expressed worries about the reliance on parental honesty in reporting results. Certain parents may lack objectivity when assessing their own child’s vision. The accuracy and reliability of visual acuity readings can be ensured by using virtually supervised at-home vision testing with a healthcare practitioner in circumstances when the results are inconsistent or do not correlate with readings obtained in traditional in-clinic settings. Participants expressed concern about the effect of external variables on results accuracy. However, differences in screen brightness on laptops and varied room illumination levels in patients’ homes have a minor impact on visual acuity assessments (Bhorade *et al*., 2013). Both factors will have no substantial effect on acuity test performance (Ritchie *et al*., 2021).

A common concern raised by participants was the accuracy of digital vision testing. While studies have shown that app-based visual acuity (VA) measurements can be equivalent to standard hospital charts (Allen *et al*., 2021; Bastawrous *et al*., 2015; Milling *et al*., 2015; Painter, Hamilton and Livingstone, 2023; Ritchie *et al*., 2021), clinical validation remains limited (Allen *et al*., 2021; Yeung *et al*., 2019).

Several studies indicate that remote testing by parents or orthoptists can achieve high repeatability compared to in-clinic assessments (e.g., Allen *et al*., 2021; Bellsmith *et al*., 2022; Thirunavukarasu *et al*., 2022; Xian *et al*., 2023). However, others report that at-home testing may slightly underestimate children’s vision when compared to in-clinic testing (Adyanthaya and Abhilash, 2021; Chen *et al*., 2021; Siktberg *et al*., 2021). This highlights the importance of using evidence-based tools and, where possible, maintaining consistency in the test used across visits to reduce variability.

Although many participants preferred testing under professional supervision, this may not be practical in busy NHS clinics and could limit the efficiency of remote models. Research on unsupervised remote testing is mixed. While some findings suggest that parent-led VA assessments can align well with gold-standard tests (Allen *et al*., 2021; Bellsmith *et al*., 2022), others note challenges in home settings such as incorrect distances, peeking, loss of interest, or unintentional help from parents that may affect reliability (Painter *et al*., 2021; Painter, Hamilton and Livingstone, 2023; Osborne *et al*., 2023). Given time constraints on clinicians, unsupervised testing may offer a more scalable and cost-effective solution but only when using validated tools and protocols.

Many of the concerns regarding remote vision testing highlighted by our participants can be overcome by advances in technology. The future in application-based home vision testing will allow for automatic target scaling with distance sensing features (Painter, Hamilton and Livingstone, 2023) holding the potential for enhanced accuracy of remote visual acuity testing thereby reducing the disparity between home and clinic standards (Painter, Hamilton and Livingstone, 2023). The future of remote vision testing has the potential to improve the way NHS uses its resources, freeing up in-person appointments for children who require them and simultaneously motivating parents to be more involved in their child’s eye care (Allen *et al*., 2021).

## Limitations

Some limitations were observed. Participants in the study were predominantly in their late 30s to early 40s, thereby excluding both younger and older parents. A significant gender imbalance was observed, with 67% of participants being female. Future research should aim for a more balanced gender representation. Most participants were employed in sectors such as education, healthcare, business, and customer service. individuals from other professional backgrounds, including stay-at-home parents were not represented in the study. Additionally, the majority (83%) of participants had two children, which limits the applicability of findings to parents with only one child or larger families. Participants generally belonged to middle- to high-income socio-economic groups and were recruited through a primary school newsletter in Buckinghamshire. This recruitment method restricted the geographic and ethnic diversity of the sample. All participants had taken their child for an eye test, and none reported regular use of hospital eye care services. In future studies researchers should aim to include a broader demographic and a wider range of eye care experiences.

## Conclusion

Overall, participants expressed concerns about factors that could compromise the reliability of remote vision testing, which in turn affected their trust in the method. However, they also acknowledged several potential benefits, including reduced travel, lower costs, minimal disruption to school or work, and greater flexibility. To address their concerns, participants suggested mitigations such as incorporating professional support when using the application.

Children’s age, personality, and attention span were seen as key factors influencing the effectiveness of remote testing. Younger or less cooperative children posed challenges, while older or more engaged children were considered better suited for at-home assessments.

Despite the convenience of remote testing, participants consistently preferred professional involvement to ensure accuracy, provide reassurance, and offer follow-up support. The frequency of hospital appointments further motivated interest in remote options, with many favouring app-based testing when paired with virtual consultations.

Future research should develop parent training programmes for at-home vision testing. Enhancements such as automatic eye movement scaling and distance detection in apps could improve accuracy. Broader studies across diverse demographics are needed to better understand parental perspectives across ages, professions, and regions.

This study lays the foundation for future mixed-methods research using online surveys and interviews to explore links between parental views, demographics, socio-economic factors, and children’s remote versus in-clinic visual acuity results. Findings will guide future research on the acceptance of virtual supervision versus parent-led remote vision testing models.

## Additional Files

The additional files for this article can be found as follows:

10.22599/bioj.541.s1 Supplementary File 1. Thematic Coding Framework and Participant Quotes from Qualitative Analysis.

10.22599/bioj.541.s2 Supplementary File 2. Focus group topic guide.
