# Supplementary material for: Exploring Parental Views of Remote Vision Testing in Children
Source: Br Ir Orthopt J. 2026 Jul 6;22(1):139–49. doi: 10.22599/bioj.541 (PMC13361081; doi:10.22599/bioj.541)
Supplement: Supplementary File 1. — Thematic Coding Framework and Participant Quotes from Qualitative Analysis. [file bioj-22-1-541-s1.pdf]

Supplementary file 1

Thematic Coding Framework and Participant Quotes from Qualitative Analysis

Theme 1: Navigating trust in remote vision testing

Subtheme	Participants Quotes
Parental engagement and trustworthiness of themselves or the app	Participant 5: “I would find it quite difficult to control all the environmental factors. At home, if you have got a set level of overhead lighting.”<br>Participant 6: “It depends on if the parents’ is doing it properly, they might be doing it at the wrong distance then the results are not accurate.”<br>Participant 5: “It depends on how confident you are because some people might not be as tech savvy as others.”<br>Participant 12: “I'd be a little bit reassured if the app was created and developed by healthcare professionals.”<br>Participant 4: “I'd rather get everything checked. It's like having a full MOT… you don’t know if there was an underlying issue behind there that you're not picking up.”<br>Participant 2: “You would completely be dependent on a parent's honesty… some parents could fabricate… I think bias could play a part.”
Potential benefits of at-home testing	Participant 3: “For someone who has to go into hospital for vision checks every six or eight weeks… it saves them time, in the journey and waiting in the hospital.”<br>Participant 9: “It would be helpful for working parents and so do not have to take too much time off work. Save on cost of travelling to appointment.”

Theme 2 – The impact of child demographics

Subtheme	Participation Quotes
Influence of child demographics on remote testing	Participant 9: “Some children will respond better to an outsider than they would with their parents, every child is different.”<br>Participant 5: “Kids really love new things, so they would see it as something new and fun to try.”<br>Participant 5: “my two-year-old if the test involved letters and numbers, he'd be fidgety.” Compared to “my four-year-old would find it interesting like oh this is cool, something a bit different.”<br>Participant 12: “Using an app just to double check your child’s vision with a professional online. It is effective, depending on the child's age and how cooperative the child is.”<br>Participant 11: “sometimes children lie, and they are like my vision's not great, mum. So, it's good just to check it yourself at home.”<br>Participant 7: “it’s the personalities and it's also the ability of the child themselves, which are variable.”<br>Participant 2: “I would give it a go, with a child that I know would be able to sit still and do it”.

Theme 3 - Desire for professional support

Subtheme	Participants Quotes
Desire for professional guidance	Participant 12: "I just think if this app is going to be as effective as it should be, then it should eliminate as much error. Someone who is a professional themselves and has that qualification overlooking while using the platform."<br>Participant 9: "I would second guess myself if I was doing a vision test on my child just because I'm not a professional."<br>Participant 6: "I will still be needing the validation from the healthcare professionals so that I know that what I'm doing is right for my child".<br>Participant 9: "I do like the idea if I did the test and then I was able to have a follow up with a professional to see if our scores matched.<br>Participant 6: "I'm going to rely on the professional opinion and if there any gaps, you know they can always refer me onwards to go to the hospital for a face-to-face appointment."
Desire for gold standard testing	Participant 5: "I might be swayed a little bit because the professional is doing it online with me. I still would prefer to go to the hospital to get it checked."<br>Participant 10: "I would still be a nervous and anxious parent, and I would want to bring him in for a face-to-face appointment."

Theme 4 – Digital enablers and barriers

Subtheme	Participants Quotes
Technical barriers and need for support	Participant 5: "I'm all about innovations and technology, it has advanced so much now, it is a reliable option."<br>Participant 10: "It does depend individually, because some people are very good with technology and some people are very good at following instructions. So, in that case, a lot of people will find it quite easy to use the app if there is a video with instructions".<br>Participant 10: "I think if it's a technical thing or if you're just not confident about using the equipment, or you want to know the distance that it should be set at, it would be good to speak to someone just to reinforce everything."<br>Participant 7: *"I'd be happy to use it just to check if everything's okay, but if my child already had an issue, I'd want a professional to keep an eye on it."*
Equity and Access Considerations	Participant 6: "Interruption with our internet connection can sometimes be a hindrance so it could waste the clinician's time. If you live in a rural location as opposed to an urban location, these are factors to consider with internet connections. It would create inequality to access depending on their location."

Theme 5 - Appointment frequency influences desire for remote testing

Subtheme	Participant Quotes
Preference for virtual health professional led at home testing	Participant 5: “I might not be able to get to the hospital with 3 monthly intervals. Using the app, I can constantly kind of check and monitor.”<br>Participant 12: “It would be convenient to have the concept of being able to do it through an app and then having that confirmed by the healthcare professional online.”<br>Participant 11: “If you are an anxious parent as well, if I'm worried that my child's vision is getting worse, then I can just check myself at home and see if it's deteriorating”.<br>Participant 3: “If my child had to come into hospital for vision checks repeatedly every six or eight weeks. I wouldn't personally mind doing this online with a health professional”.
