# Supplementary material for: Exploring Parental Views of Remote Vision Testing in Children
Source: Br Ir Orthopt J. 2026 Jul 6;22(1):139–49. doi: 10.22599/bioj.541 (PMC13361081; doi:10.22599/bioj.541)
Supplement: Supplementary File 2. — Focus group topic guide. [file bioj-22-1-541-s2.pdf]

# Supplementary File 2

## Focus group topic guide

| |
|---|
| Hello everyone.<br>Thank you for taking the time to talk to me today.<br>I am……………… (gives a brief introduction)<br><br>Have you all had a chance to read through the patient information leaflet and guidance sheet for today's focus group?<br><br>Your input as a parent/guardian surrounding this topic is very important. This focus group aims to explore your views on having your child's vision tested at home either by yourself or via a clinician on a platform like Microsoft Teams or Zoom.<br><br>This discussion should last for approximately 90 minutes and will be recorded.<br><br>You know that anything you say will be kept entirely confidential, and you will not be identified in any form or way. We can stop at any time and understand that you are happy for the discussion to be recorded? |
| "*Before we begin, I would like to review some of the ground rules for the discussion, which are as follows*; "<br>All participants must listen to each other and not interrupt or talk over one another.<br>All participants must respect each other's opinions and comments made.<br>Participants may ask questions but remember the facilitator is not here to share their opinion or indulge in the discussion.<br>Participants should not make derogatory remarks about the topics, or the environment participants work in.<br>Participants should criticise constructively if there is a disagreement with an opinion.<br>It is fine to have a difference in opinion. However, please do respect each other's opinions.<br>Participants should not ask personal questions.<br>All participants should contribute.<br>No participant should dominate the discussion.<br>Everything should remain confidential and should not be discussed outside the group.<br>GET VERBAL CONSENT TO START RECORDING. TURN ON THE RECORDING IN ZOOM/TEAMS. |
| Icebreaker *"I would like you all to one by one take turns and introduce yourself to each other. Please tell us your first name only and how many children you look after and their ages."* |
| Today we are here to learn about you views surrounding remote vision testing.<br><br>Topics to be discussed<br><br>**How confident would you feel with assessing your child's vision remotely using an application which you have downloaded onto a device such as an iPad or tablet? After watching a video and reading some instructions you would be requested to measure your child's vison and then e-mail their anonymized results to the hospital?**<br>*General discussion on how comfortable the group feel* |

**Probes to be used as needed**

What feelings about remote vision testing depend on the level of child’s cooperation?
For example, their age
For example, their personality type, shy or listens to authority, becomes bored easily
For example, relationship with child- parent/guardian has no problems in giving medicine or carrying out homework

What feelings about remote vision testing depend on the level of parental/guardian skills set?
For example, ability with technology, downloading application, photographing results, emailing to HES, previous experience with carrying out medical tests

What feelings about remote vision testing depend on parental motivation?
For example
Accuracy or reliability with either test
Anxious about responsibility if they were to test
Flexibility increases with remote testing as it can be carried out at best time for child/family
Can practice test with child at home so that they understand
Child is more likely to listen to a clinician
Second eye can be tested at a different time
Child might be less anxious at home
Desire to talk to a professional clinician
Familiarity with technology
Views on cost
Views about speed- may either like a more thorough appointment or prefer most efficient appointment
Worries about missing school
Distance from hospital
Difficulties with transportation
Digital poverty-lack of iPad-tablet
Data not secure
Worries about equipment
Other similar probes

**Is there anything that can be done to overcome any worries about remote vision testing at home using an application which is downloaded onto tablet? For example, having a number to ring or an e-mail to send if they have concerns or worries, being able to state not reliable in results or rate their accuracy**

**How confident would you feel with a qualified clinician measuring your child’s vision remotely whilst you both remain at home? You would login into a platform such as Zoom or Microsoft Teams with your iPad, tablet or computer. A qualified clinician would then meet you online at a specified date and time and talk you both through the entire test?**

Use probes as above as needed

Participants will now be asked about the advantages and disadvantages of remote vision testing.

**What are the advantages of remote vision testing**? If no one answers then use prompts such as more flexible, no/less time off work

Expand on each advantage that is brought up and seek everyone’s thoughts

| |
|---|
| **What are the disadvantages of remote vision testing**? If no one answers then use prompts such as not reliable/accurate, you may make a mistake<br>Expand on each disadvantage that is brought up and seek everyone's thoughts<br><br>The facilitator summarises the advantages and disadvantages that have been discussed<br><br>**Have your views about remote vision testing changed since COVID-19?**<br>*General group discussion*<br><br>**Have you ever had remote healthcare from another medical professional such as a nurse or a GP for your child? How did you feel about that/those appointments?**<br>*General group discussion*<br><br>**How often do you feel that you would like to use remote vision testing for your child?**<br>*General group discussion*<br><br>**Given a choice which would you prefer carrying out the vision screening by yourself or with a qualified clinician who is carrying out the vision testing via a platform such as Microsoft Teams or Zoom?**<br>*General group discussion*<br><br>**Would anything change your opinion? For example, if you had initially practiced the procedure with a qualified clinician?**<br>*General group discussion* |
| The facilitator will ask the participants if they would like to add any more factors that come to mind which were not identified previously in the discussion<br>"*Does anyone have any final thoughts about the advantages and disadvantages discussed or anything you may feel has been missed out but is relevant*?" |
| "Thank you very much for taking the time and sharing your opinions with us. We hope you enjoyed the discussion today. |
